# Shape Prediction of the Sheet in Continuous Roll Forming Based on the Analysis of Exit Velocity

**DOI:** 10.3390/ma14185178

**Published:** 2021-09-09

**Authors:** Jia-Xin Gao, Qing-Min Chen, Li-Rong Sun, Zhong-Yi Cai

**Affiliations:** 1 College of Materials Science and Engineering, Jilin University, Changchun 130025, China; gaojx1106@163.com (J.-X.G.); sunlr92@163.com (L.-R.S.); 2 Roll Forging Research Institute, Jilin University, Changchun 130025, China

**Keywords:** continuous roll forming, double curvature surface, exit velocity, longitudinal curvature radius, numerical simulation

## Abstract

Continuous roll forming (CRF) is a new technology that combines continuous forming and multi-point forming to produce three-dimensional (3D) curved surfaces. Compared with other methods, the equipment of CRF is very simple, including only a pair of bendable work rolls and the corresponding shape adjustment and support assembly. By controlling the bending shapes of the upper and lower rolls and the size of the roll gap during forming, double curvature surfaces with different shapes can be produced. In this paper, a simplified expression of the exit velocity of the sheet is provided, and the formulas for the calculation of the longitudinal curvature radius are further derived. The reason for the discrepancy between the actual and predicted values of the longitudinal radius is deeply discussed from the perspective of the distribution of the exit velocity. By using the response surface methodology, the effects of the maximum compression ratio, the sheet width, the sheet thickness, and the transverse curvature radius on the longitudinal curvature radius are analyzed. Meanwhile, the correction coefficients of the predicted formulas for the positive and negative Gaussian curvature surfaces are obtained as 1.138 and 0.905, respectively. The validity and practicability of the modified formulas are verified by numerical simulations and forming experiments.

## 1. Introduction

With the rapid development of the manufacturing and construction industries, the market has higher requirements for the production technology of 3D sheet materials. While ensuring the production quality, it is also necessary to pursue lower manufacturing costs. The mode of production has also changed from mass production to single piece or small batch production. Therefore, the traditional mold forming and hydroforming methods are no longer applicable due to the single production type and the high mold maintenance costs. The market urgently requires a new method to produce 3D sheet metals.

**Figure 1:**
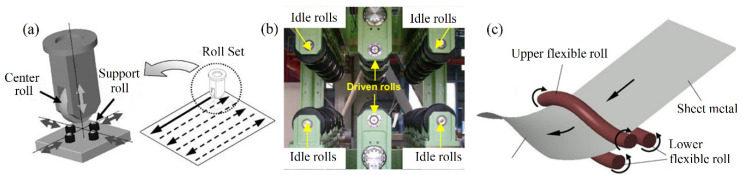
Schematic diagram of continuous forming technology of 3D surfaces: (**a**) FIRF process. With permission from Reference [6], copyright (2003) Elsevier; (**b**) LARS process. With permission from Reference [8], copyright (2010) Elsevier; (**c**) CFF process. With permission from Reference [11], copyright (2012) Elsevier.

Many researchers have tried to introduce the ideas of flexible forming and continuous forming into the traditional forming method [1,2,3,4], which not only achieves the application of one mold to manufacture 3D sheets with different shapes but also solves the problem that the size of the shaped part is limited by the mold size. Yamashita and Yamakawa [5] realized the double bending deformation of sheet metal with an entrance roll, exit rolls, and a pair of flexible rolls supported by bearings. However, because of the discontinuity of the flexible rolls, the forming quality is poor. Yoon and Yang [6,7] put forward a flexible incremental roll forming (FIRF) method, which uses one upper center roll and two pairs of lower support rolls as forming tools to gradually complete the deformation of sheet metal in the whole region. In order to improve the production efficiency, Shim et al. [8,9] proposed the line array roll set (LARS) process containing three pairs of rolls. In addition, Cai et al. [10,11] advanced a continuous flexible forming (CFF) method, in which the rigid rolls are replaced by the bendable rolls. Schematic diagrams of the FIRF process, LARS process, and CFF process are shown in Figure 1a–c, respectively.

In recent years, Li et al. [12,13] developed a new method named continuous roll forming (CRF), which only needs two flexible rolls to form 3D sheet metals. Cai et al. [14,15,16,17] analyzed the deformation mechanism of CRF from the mechanical point of view and gave the necessary conditions for forming. Li et al. [18,19] discussed the effects of sheet length, width, and thickness on the material utilization. In related research, they discovered that the use of differential speed rotation technology can significantly improve material utilization. Wang et al. [20] extended CRF technology so that the sheet can be bent laterally on the basis of transverse and longitudinal bending. Shape prediction is a difficult problem in the CRF process. Many scholars have explored the calculation method of longitudinal bending radius of the sheet. Yoon et al. [21] established a prediction model of longitudinal curvature radius by defining deformation vectors. For the purpose of improving the efficiency and accuracy of the prediction, Ghiabakloo et al. [22,23,24] decomposed the shape prediction into three regions, namely pre-rolling, rolling, and post-rolling, each of which was solved by an appropriate method. Park et al. [25,26] constructed a regression model and an artificial neural network model, respectively, and they proved that the regression model is suitable for convex surfaces, and the artificial neural network model performs better while producing saddle surfaces after comparison.

This article provides a novel approach for predicting the shape of the sheet in CRF based on the exit velocity. The simplified expression of the exit velocity of the sheet metal is offered, and the calculation formulas of the longitudinal curvature radius are further deduced. The reason for the difference between the actual value and the predicted value of the longitudinal curvature radius is analyzed. By means of the response surface methodology, the effects of maximum compression ratio, sheet width, sheet thickness, and transverse curvature radius on the longitudinal curvature radius are studied, and the correction coefficients of the predicted formulas for positive and negative Gaussian curvature surfaces are provided. Numerical simulations and forming experiments are carried out to verify the validity of the revised formulas.

## 2. Materials and Methods

### 2.1. Materials Used in Simulations and Experiments

The CRF process is dedicated to turning flat sheets into double curvature sheets. In the following simulations and experiments, the rectangular sheets made of Al 2024-O are used. The mechanical properties of the material are obtained by uniaxial tensile testing as follows: elastic modulus E=72.4 GPa, yield strength $\sigma_{s}=75.53\;MPa$, density $\rho =2.72\;g/cm^{3}$ and Poisson’s ratio μ=0.34. It is considered that the material obeys Mises yield criterion and conforms to the elastoplastic constitutive relationship with isotropic hardening. Table 1 shows the chemical composition of the material. The length, width, and thickness of the sheet metal are 280–300 mm, 210–270 mm and 1.5–2 mm, respectively.

### 2.2. The Principle of CRF

CRF is an efficient new method of 3D surface forming. The main forming tool is a pair of flexible upper and lower rolls. In the forming process, according to the shape of the target surface, the contours of the work rolls are adjusted by controlling the shape adjustment units evenly arranged on the rolls, so as to control the transverse bending deformation of the sheet metal. Due to the large plastic deformation in the CRF process, the effect of elastic deformation is ignored. Therefore, the transverse curvature radius of the sheet metal is the same as the radius of the roll gap centerline.

**Figure 2:**
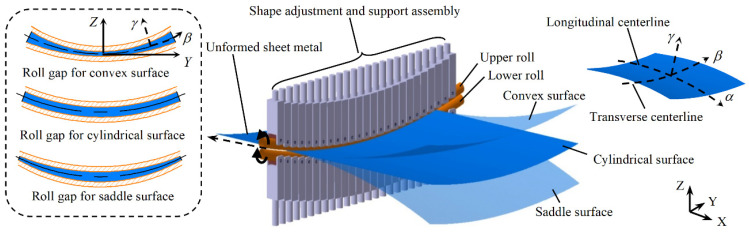
Schematic diagram of continuous roll forming.

The uneven roll gap is the key to longitudinal bending. As shown in Figure 2, if the roll gap is small in the middle and large on both sides, the sheet metal will bend with positive Gaussian curvature and form a convex-shaped surface. If the distribution of the roll gap is opposite, a saddle-shaped surface with negative Gaussian curvature will be formed. With a uniformly distributed roll gap, the forming process degenerates into ordinary roll bending, forming a cylindrical surface. Specifically, when the sheet passes through the uneven roll gap, the velocities of the exit cross-section at different positions in the transverse direction are different. Since the centerline of the roll gap is not horizontal, the difference of the velocities causes the exit cross-section to rotate in the longitudinal direction, thereby forming the longitudinal curvature of the sheet. The exit velocity of a positive Gaussian curvature surface is large in the middle and small on both sides, while that of a negative Gaussian curvature surface is small in the middle and large on both sides. The exit velocity corresponding to the cylindrical surface is the same in the transverse direction, so the sheet will not bend longitudinally. The reverse rotation of the two rolls with the same speed moves the sheet forward until the 3D surface is finally formed.

### 2.3. The Basic Equation and Some Simplifications in CRF

**Figure 3:**
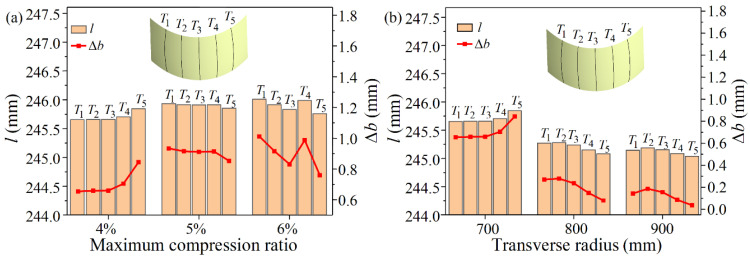
Comparison of the transverse dimensions of the sheet before and after rolling: (**a**) Under the condition of different maximum compression ratios; (**b**) Under the condition of different transverse radii.

The principle of constant volume of plastic deformation can be expressed in the rolling process as a constant volume of metal passing through any cross-section within the deformation zone per unit of time. In continuous roll forming, the metal flow volume per second through the entrance cross-section and the exit cross-section is equal, that is:

(1) $$v_{H}HB=\iint_{S}v_{1}(\beta ,\gamma )dS$$

where $v_{H}$ is the moving velocity of the metal at the entrance cross-section, H and B are the original thickness and width of the sheet, $v_{1}(\beta ,\gamma )$ is the longitudinal (X direction) velocity of the metal at the exit cross-section, β and γ are the curve parameter coordinate corresponding to the direction along the centerline of the cross-section and normal to the surface, respectively, and S is the area of the exit cross-section, which is given by

(2) $$S=\int_{-\frac{l}{2}}^{\frac{l}{2}}h(\beta )d\beta$$

where h(β) and l are the roll gap function and transverse arc length of the sheet after rolling, respectively. The compression of the sheet thickness should be converted into longitudinal elongation and transverse spread Δb, so l can be written as l=B+Δb. As the width of the rolling deformation zone is much larger than the length, the longitudinal flow of metal is much easier than the transverse flow according to the law of minimum resistance. The formula [27] given by Bakhtinov for Δb is used to evaluate the amount of transverse spread in CRF, which is denoted as

(3) $$\Delta b=1.15\frac{\Delta h}{2H}(\sqrt{R_{roll}\Delta h}-\frac{\Delta h}{2f})$$

where Δh is the sheet reduction in the thickness direction, $R_{roll}$ is the radius of the work roll, and f is the friction coefficient. It can be seen from Equation (3) that the spread mainly depends on the reduction. The larger the reduction is, the larger the spread is. In CRF, the compression ratio λ=Δh/H of sheet metal will not exceed 15%, and if the sheet size is 280 mm × 245 mm × 2 mm, the reduction will not exceed 0.3 mm. While setting f=0.2 and $R_{roll}=5\;mm$, the maximum spread obtained by Equation (3) is 0.0409 mm. The transverse relative spread η=Δb/B=0.017%, which is far less than the relative reduction 15%, indicating that the spread can be ignored. It is further illustrated by numerical simulation that the spread can be neglected. The key to CRF is that the rolls are curved, and the height of the roll gap is variable in the transverse direction. Here, different maximum compression ratios and transverse curvature radii are set to compare the transverse dimensions of the sheet before and after rolling (B and l), as shown in Figure 3. It should be noted that the minimum compression ratio at the edge of the sheet is set to 0 for the positive Gaussian curvature surface.

In Figure 3, $T_{1}\sim T_{5}$ are the positions used for comparison. It can be seen that the transverse arc lengths of the sheet after rolling are essentially the same, with a difference of less than 1 mm. Compared to the original width of 245 mm, the transverse dimension after rolling increases slightly. The largest increase of transverse dimension is 1.011 mm at $T_{1}$ when the maximum compression ratio is 6%. The transverse relative spread η is 0.413%, which is small enough to be neglected compared with λ=6%. Therefore, whether through theoretical calculation or numerical simulation, it can be concluded that the spread can be ignored in CRF, and l=B is determined.

**Figure 4:**
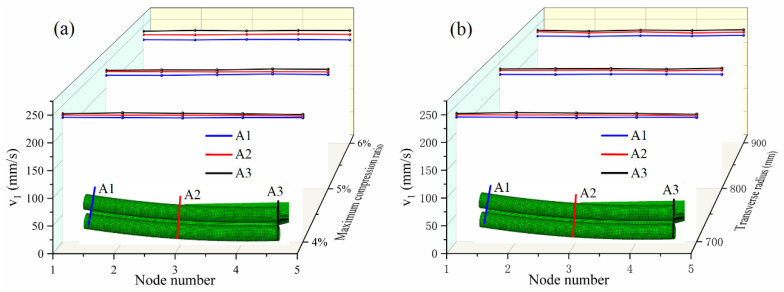
Longitudinal exit velocity of the nodes on the normal line of sheet metal: (**a**) Under the condition of different maximum compression ratios; (**b**) Under the condition of different transverse radii.

Due to the thinness of the sheet, it is assumed that the longitudinal exit velocities of the metal particles which are on the normal line (γ direction) of the middle surface are the same and that the longitudinal exit velocity of the cross-section can be expressed by the exit velocity of the particle at the centerline (β-line). This assumption is verified by numerical simulations. The sheet is taken with 4 elements in the γ direction, and hence, there are 5 nodes. Under the conditions of different maximum compression ratios and transverse curvature radii, the longitudinal exit velocities of different nodes on the normal line of the middle surface of the sheet are extracted, as shown in Figure 4. Owing to the symmetry of the structure, only 1/2 of the finite element model is taken for the simulation in order to save the calculation time. A1 and A3 are the normal lines of the middle surface corresponding to the minimum and maximum compression ratios. Nodes 1 and 5 are the nodes in contact with the upper and lower rolls, respectively. As seen in Figure 4, the longitudinal exit velocities of the five nodes are essentially the same on either normal line. Increasing the maximum compression ratio or transverse curvature radius can increase the velocity difference between the nodes on A1 and A3. Hence, in continuous roll forming, the longitudinal exit velocity $v_{1}$ does not change with γ, which can be expressed as $v_{1}(\beta ,\gamma )=v_{1}(\beta )$.The roll gap function h(β) can be denoted by the compression ratio function λ(β) as

(4) h(β)=H[1−λ(β)].

Substituting Equations (2) and (4) together with l=B into Equation (1), there is

(5) $$v_{H}B=\int_{-\frac{B}{2}}^{\frac{B}{2}}v_{1}(\beta )[1-\lambda (\beta )]d\beta .$$

The right-hand side of Equation (5) contains the unknown complex function of β. It is not possible to derive an intuitive expression for $v_{1}(\beta )$, so further simplification is required. If the sheet metal is divided into small sections with transverse dimension dβ for consideration, $v_{1}(\beta )$ can be solved from Equation (5) as follows:

(6) $$v_{1}(\beta )=\frac{v_{H}}{1-\lambda (\beta )}$$

### 2.4. Derivation of Longitudinal Curvature Radius in Ideal Continuous Roll Forming

**Figure 5:**
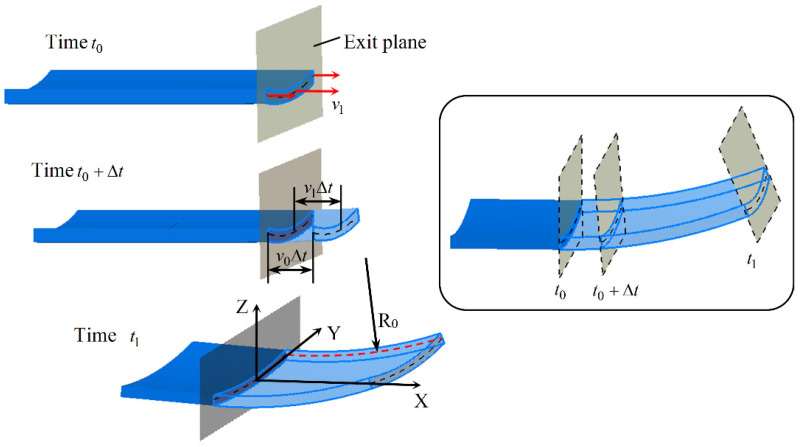
Process of ideal continuous roll forming.

In order to facilitate the derivation, the Cartesian coordinate system is used here rather than a curved coordinate system. In an ideal CRF process, adjusting the roll gap (i.e., controlling the compression ratio function) so that the longitudinal exit velocity is exactly a linear function of the Z-coordinate will cause the cross-section of the sheet to rotate around a fixed axis after exiting the roll gap and remain flat, as shown in Figure 5.

For positive Gaussian curvature surface, there is

(7) $$\frac{v_{1}\Delta t}{R_{0}{+Z}_{\delta }-Z}=\frac{v_{0}\Delta t}{R_{0}}$$

where $Z_{\delta }$ is the height difference between the center and the edge of the centerline of the cross-section, $v_{1}\Delta t$ is the displacement of the sheet in the longitudinal direction (X direction) through the time Δt, $v_{0}=v_{1}{|}_{Z=Z_{\delta }}$, and $R_{0}$ is the longitudinal curvature radius of the sheet at the position ${Z=Z}_{\delta }$. The relationship between $v_{1}$ and the Z-coordinate can be obtained from Equation (7) as

(8) $$v_{1}=-\frac{v_{0}}{R_{0}}Z+\frac{R_{0}+Z_{\delta }}{R_{0}}v_{0}.$$

The longitudinal curvature radius can be given as follows:

(9) $$R_{0}=\frac{(Z_{\delta }-Z)v_{0}}{v_{1}-v_{0}}.$$

Thus, in ideal CRF, the target double curvature surface can be formed with bending rolls by controlling the size of the roll gap so that the longitudinal exit velocity is a linear function of the Z-coordinate and adjusting the bending radius of the roll gap centerline to be the same as the transverse curvature radius of the target surface.

For a surface with negative Gaussian curvature, the center of the sheet is not compressed. The derivation process of the expression of longitudinal exit velocity $v_{1}{}^{\prime }$ is similar to that of the positive Gaussian curvature surface, and $v_{1}{}^{\prime }$ can be obtained as follows:

(10) $$v_{1}{}^{\prime }=\frac{v_{0}{}^{\prime }}{R_{0}{}^{\prime }}Z+v_{0}{}^{\prime }.$$

Hence, there is

(11) $$R_{0}{}^{\prime }=\frac{v_{0}{}^{\prime }Z}{v_{1}{}^{\prime }-v_{0}{}^{\prime }}$$

where $v_{0}{}^{\prime }=v_{1}{}^{\prime }{|}_{Z=0}$ and $R_{0}{}^{\prime }$ is the longitudinal curvature radius of the negative Gaussian curvature surface at the position Z=0.

## 3. Results and Discussion

### 3.1. Generation and Analysis of Forming Errors

**Figure 6:**
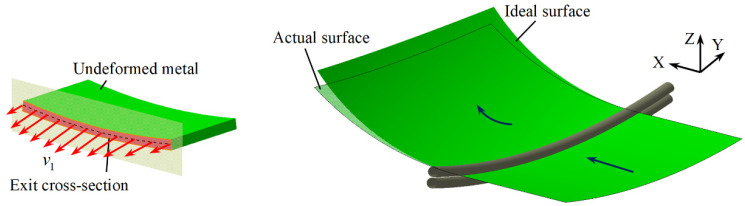
Positive Gaussian curvature surface formed by CRF.

Numerical simulations are carried out to form positive and negative Gaussian curvature surfaces with the sheet size of 300 mm × 270 mm × 1.5 mm, a transverse curvature radius of 800 mm, and a maximum compression ratio of 0.07. The difference between the two types of double curvature surfaces is that the maximum compression of a positive Gaussian curvature surface is at the center of the sheet, whereas the maximum compression of a negative Gaussian curvature surface is at the edge of the sheet. The simulated results reveal that the longitudinal curvature radius is not consistent with the value calculated by (9) or (11), which is due to the fact that the actual exit velocity does not follow the expected linear distribution. Figure 6 is a schematic diagram of forming a positive Gaussian curvature surface. The actual simulated longitudinal curvature radius is larger than the ideal calculated result.

**Figure 7:**
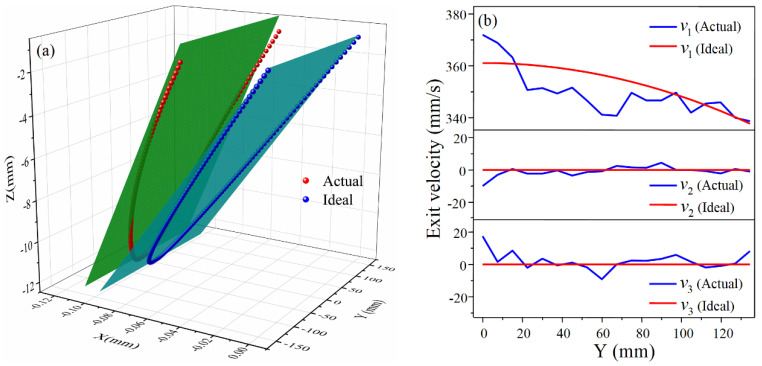
Node distribution of exit cross-section centerline and exit velocity for positive Gaussian curvature surface: (**a**) Node distribution; (**b**) Exit velocity in three directions.

In order to deeply analyze the influence of the distribution of exit velocity on the longitudinal curvature radius, the node coordinates of the centerline of the exit cross-section and the exit velocities are extracted as shown in Figure 7, and $v_{1}$, $v_{2}$, and $v_{3}$ are the exit velocities in the X, Y, and Z directions, respectively.

As can be seen from Figure 7a, in ideal CRF, the cross-section (obtained by calculation) remains planar throughout the forming process as it rotates around a fixed axis, i.e., all nodes lie on the fitted plane. However, in practice, due to the complexity of the metal flow, the distribution of longitudinal exit velocity is not in a standard linear form so that the actual distribution of exit nodes deviates to some extent from the fitted plane. The inclination degree of the actual fitted plane is less than that of the ideal fitted plane, indicating that the longitudinal curvature of the sheet metal in the actual forming is less than the ideal value, which is consistent with the comparison of the forming results. This phenomenon can be well explained in Figure 7b. Ideally, the metal on the exit cross-section flows only in the X direction after the upper roll is pressed down, and the longitudinal exit velocity is a linear function of the Z-coordinate with velocities in the Y and Z directions being zero. In the actual simulation results, $v_{1}$ is much larger than $v_{2}$ and $v_{3}$, both of which fluctuate around zero, demonstrating that it is reasonable to neglect $v_{2}$ and $v_{3}$ at the exit cross-section in CRF. As for $v_{1}$, the value at the edge of the sheet is the smallest, which is basically consistent with the ideal value. At most other locations, $v_{1}$ is less than the ideal value, resulting in the decrease of the velocity difference of the exit cross-section, which is reflected in Equation (9) as a reduction in the denominator, so the actual formed longitudinal curvature radius is larger than the ideal predicted value.

**Figure 8:**
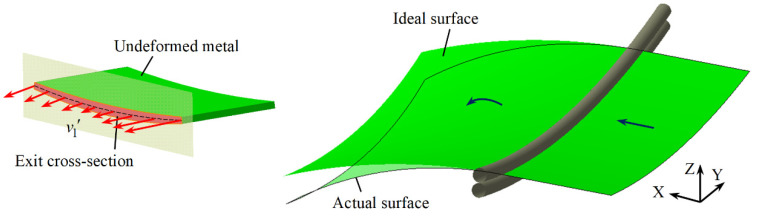
Negative Gaussian curvature surface formed by CRF.

**Figure 9:**
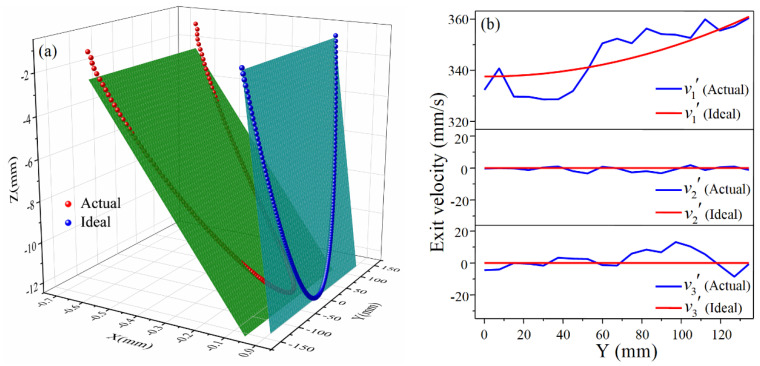
Node distribution of exit cross-section centerline and exit velocity for negative Gaussian curvature surface: (**a**) Node distribution; (**b**) Exit velocity in three directions.

Figure 8 shows the forming situation for a negative Gaussian curvature surface, from which it can be seen that the actual longitudinal bending radius is smaller than the ideal predicted radius. The coordinates of the exit nodes and the exit velocities of the negative Gaussian curvature surface are extracted for analysis, as shown in Figure 9. It is evident from Figure 9a that the exit nodes in the ideal case are distributed on a plane; that is to say, the cross-section remains flat during the forming process and has a tendency to bend downwards. Some nodes on the actual exit cross-section are slightly deviated from the fitted plane, and the inclination of the actual fitted plane is greater than that of the ideal fitted plane, illustrating that the actual longitudinal curvature of the negative Gaussian curvature surface is larger than the predicted value. Figure 9b is the actual distribution of exit velocities in the three directions for the negative Gaussian curvature surface. Similar to the positive Gaussian curvature surface, $v_{2}{}^{\prime }$ and $v_{3}{}^{\prime }$ corresponding to the Y and Z directions fluctuate around zero, which are much smaller than $v_{1}{}^{\prime }$ and can therefore be ignored. The longitudinal exit velocity $v_{1}{}^{\prime }$ near the edge of the sheet is greater than the ideal value, while $v_{1}{}^{\prime }$ near the center of the sheet is smaller than the ideal value. This increases the velocity difference of the exit cross-section and leads to an increase in the denominator in Equation (11), so the actual formed longitudinal radius of the negative Gaussian curvature surface is smaller than the ideal predicted radius.

### 3.2. Influence of Various Factors on Longitudinal Radius Studied by Response Surface Methodology

The response surface methodology can be used to evaluate the nonlinear relationship between indexes and factors. In this section, the influence of maximum compression ratio (X_1_), sheet width (X_2_), sheet thickness (X_3_), and transverse curvature radius (X_4_) on the longitudinal curvature radius is studied based on Box–Behnken design (BBD). Design expert software is used for response surface analysis. The level setting of factors and the test results are shown in Table 2 and Table 3, respectively.

In Table 3, $Y_{c0}$ and $Y_{s0}$ are the curvature radii of the longitudinal centerline of the positive and negative Gaussian curvature surfaces calculated by Equations (9) and (11), respectively. $Y_{c1}$ (mm) and $Y_{s1}$ are the corresponding simulated radii. $\Delta Y_{c}$ and $\Delta Y_{s}$ represent the absolute errors.

**Figure 10:**
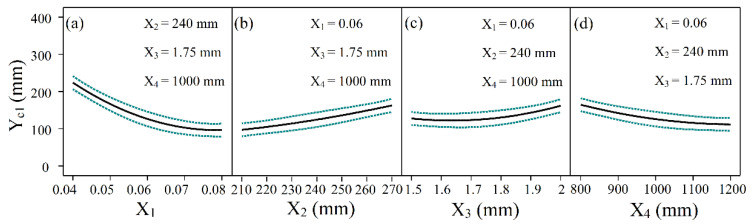
Effect of various factors on the longitudinal radius of positive Gaussian curvature surface: (**a**) Maximum compression ratio; (**b**) Sheet width; (**c**) Sheet thickness; (**d**) Transverse curvature radius.

**Figure 11:**
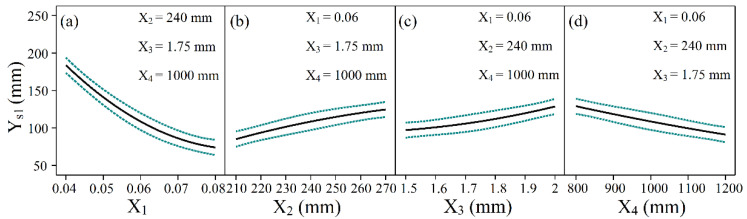
Effect of various factors on the longitudinal radius of the negative Gaussian curvature surface: (**a**) Maximum compression ratio; (**b**) Sheet width; (**c**) Sheet thickness; (**d**) Transverse curvature radius.

It is apparent from Table 3 that the longitudinal curvature radius of the negative Gaussian curvature surface is much smaller than that of the positive Gaussian curvature surface when all factors are the same. Figure 10 and Figure 11 illustrate the effect of each factor on the longitudinal curvature radius for positive and negative Gaussian curvature surfaces respectively, with the green dashed line indicating the confidence interval (95%). It is observed that the maximum compression ratio and the transverse curvature radius affect the longitudinal curvature radius negatively, while the sheet width and thickness have positive effects. Equations (9) and (11) can explain these trends. In Equation (9), let Z=0, and in Equation (11), let ${Z=Z}_{\delta }$, then the formulas can be solved via the difference of longitudinal exit velocity and difference of height ($Z_{\delta }$) between the center and edge of the centerline of the exit cross-section. The larger the maximum compression ratio is, the greater the difference of longitudinal exit velocity is, resulting in the formation of a smaller longitudinal curvature radius. Under the same other conditions, if the width of the sheet increases, $Z_{\delta }$ will increase, thereby increasing the longitudinal curvature radius. Enlarging the transverse curvature radius can have the same effect as decreasing the width, which means that $Z_{\delta }$ is reduced so that the longitudinal curvature radius is decreased. As for the thickness, if the thickness is increased, the bending resistance of the sheet metal will be enhanced, and the longitudinal curvature radius becomes larger.

## 4. Correction and Verification of the Formulas for Longitudinal Curvature Radius

### 4.1. Correction of the Formulas

Since the longitudinal exit velocity does not conform to a standard linear distribution, the longitudinal radius of the positive Gaussian curvature surface is larger than the calculated value of Equation (9), and the longitudinal radius of the negative Gaussian curvature surface is smaller than the calculated value of Equation (11). Therefore, it is considered to increase the correction coefficient on the basis of the original formulas, so that the formulas can make the prediction more accurate. The longitudinal curvature radii of the 27 sets of tests obtained by the response surface method are compared with the calculated values, and it is found that the ratio between the two is close to a constant. Thus, the average value of the ratio is taken as the correction coefficient. After calculation, when the maximum compression ratio is 0.04–0.08, the sheet width is 210–270 mm, the sheet thickness is 1.5–2 mm, and the transverse curvature radius is 800–1200 mm, the correction coefficient for the positive Gaussian curvature surface is 1.138, and that for the negative Gaussian curvature surface is 0.905. The formulas for calculating the longitudinal curvature radius of the positive and negative Gaussian curvature surfaces after the correction are as follows:

(12) $$R_{0}=\frac{1.138(Z_{\delta }-Z)v_{0}}{v_{1}-v_{0}}$$

and

(13) $$R_{0}{}^{\prime }=\frac{0.905v_{0}{}^{\prime }Z}{v_{1}{}^{\prime }-v_{0}{}^{\prime }}.$$

### 4.2. Verification of the Revised Formulas by Numerical Simulations

**Figure 12:**
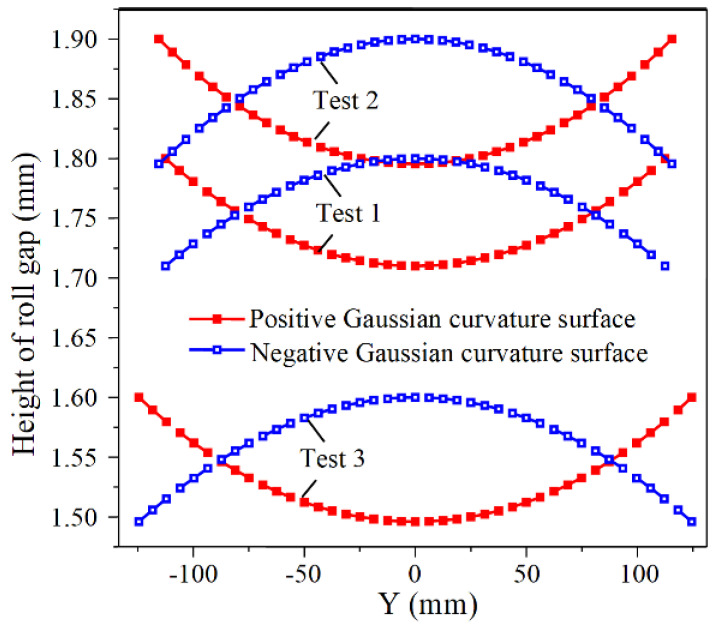
Distribution of the roll gap.

**Figure 13:**
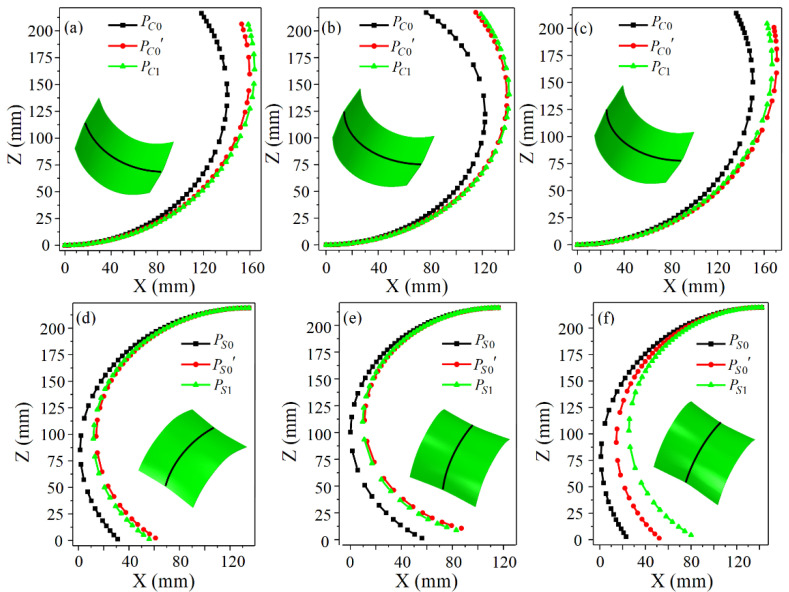
Comparison of the profiles of the longitudinal centerline: (**a**) Test 1—positive Gaussian curvature surface; (**b**) Test 2—positive Gaussian curvature surface; (**c**) Test 3—positive Gaussian curvature surface; (**d**) Test 1—negative Gaussian curvature surface; (**e**) Test 2—negative Gaussian curvature surface; (**f**) Test 3—negative Gaussian curvature surface.

Three sets of numerical simulations are used to verify the validity of the revised formulas. The specific arrangements are shown in Table 4. Figure 12 is the distribution of the roll gap. It can be seen that the roll gap function of the positive Gaussian curvature surface is a concave function, and that of the negative Gaussian curvature surface is a convex function. The profiles of the longitudinal centerlines of the double curved surfaces are extracted after the simulation and compared to the calculated profiles, which is shown in Figure 13.

$P_{C0}$, $P_{C0}{}^{\prime }$, and $P_{C1}$ are the calculated profile before correction, the calculated profile after correction, and the simulated profile of the longitudinal centerline for the positive Gaussian curvature surface, respectively, and those for the negative Gaussian curvature surface are represented by $P_{S0}$, $P_{S0}{}^{\prime }$, and $P_{S1}$. Obviously, before correction, the calculated radius of the positive Gaussian curvature surface is smaller than the simulated radius, and that of the negative Gaussian curvature surface is greater than the simulated radius. For all double curved surfaces, the corrected calculated profiles are extremely close to the simulated profiles, proving that the corrected formulas are valid.

### 4.3. Verification of the Revised Formulas by Forming Experiment

**Figure 14:**
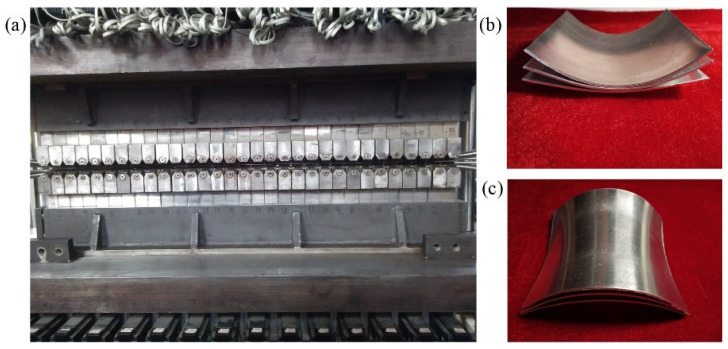
Experimental equipment of CRF and the formed parts: (**a**) Equipment; (**b**) Positive Gaussian curvature parts; (**c**) Negative Gaussian curvature parts.

The experimental equipment and the formed parts of CRF are shown in Figure 14. The shapes of the upper and lower work rolls are adjusted by 31 control units, respectively. In the working process, the rolls can maintain their shapes and rotate around their own bending axes. The outer diameter of each work roll is 10 mm. Figure 14b,c show that the quality of the formed parts is good, and there are no defects such as wrinkles and dimples.

**Figure 15:**
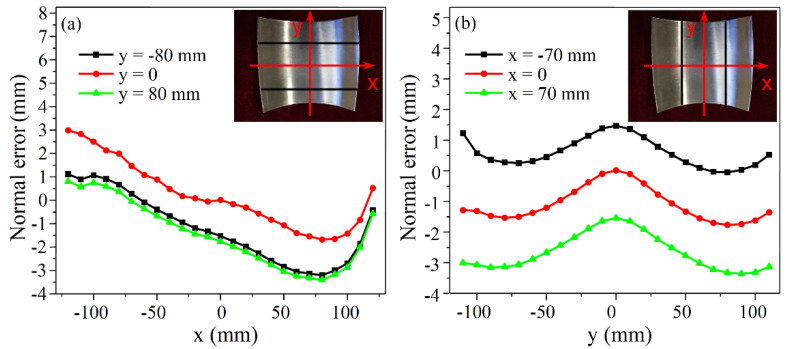
Normal error of the profile curves for positive Gaussian curvature surface: (**a**) Longitudinal; (**b**) Transverse.

**Figure 16:**
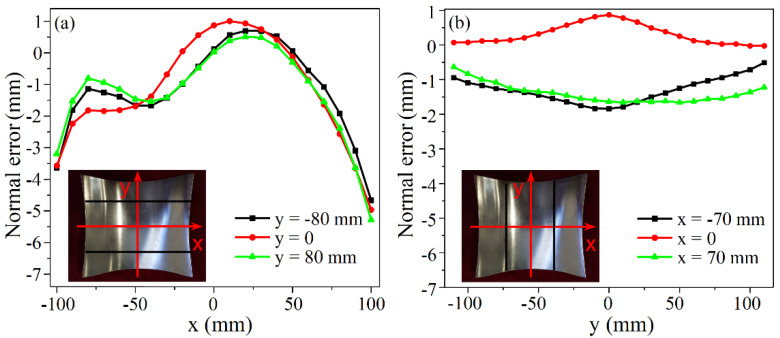
Normal error of the profile curves for negative Gaussian curvature surface: (**a**) Longitudinal; (**b**) Transverse.

Set X_1_ = 0.05, X_2_ = 225 mm, X_3_ = 1.8 mm, X_4_ = 950 mm, and the longitudinal curvature radii of the positive and negative Gaussian curvature parts predicted by the formulas after correction are 159.75 mm and 120.99 mm, respectively. The point cloud data of the experimental part are obtained by the binocular stereo vision measurement equipment. By comparing the surface formed by the point cloud with the predicted curved surface using the reverse engineering software Geomagic Qualify, the three-dimensional normal errors of the longitudinal and transverse profiles are gained as shown in Figure 15 and Figure 16. The longitudinal profiles are taken as y = 0 and y = ±80 mm, and the transverse profiles are obtained as x = 0 and x = ±70 mm. The normal error range of the longitudinal profiles of the part with positive Gaussian curvature is from −3.401 to 2.990 mm, and that of the part with negative Gaussian curvature is from −5.278 to 1.006 mm. As for the transverse profiles, the absolute values of the normal errors are within 3.5 mm for the positive Gaussian curvature part and within 2 mm for the negative Gaussian curvature part. The above errors are small enough to be considered within the acceptable range, further demonstrating the applicability of the corrected formulas.

### 4.4. Extension of the Revised Formulas for Different Materials

**Figure 17:**
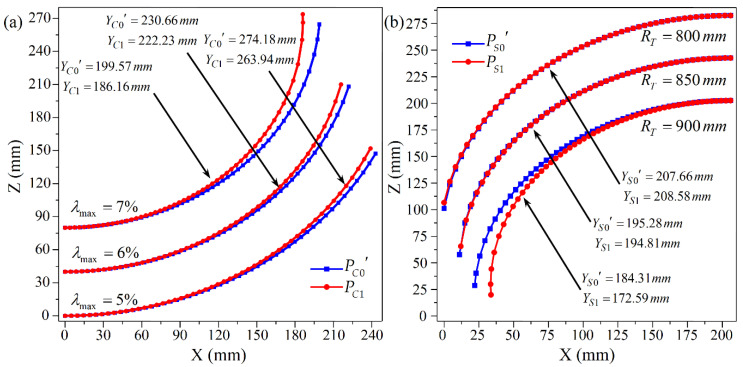
Comparison of the profiles of the longitudinal centerline for 08 Al sheets: (**a**) Positive Gaussian curvature surface; (**b**) Negative Gaussian curvature surface.

In order to study the applicability of the revised formulas to other materials, numerical simulations for CRF are carried out using 08 Al instead of Al 2024-O. The mechanical properties of 08 Al are as below: density $\rho =7.845\;g/cm^{3}$, elastic modulus E=207 GPa, yield strength $\sigma_{s}=91.294\;MPa$, and Poisson’s ratio μ=0.29. The size of the sheet is 300 mm × 270 mm × 2 mm. For positive Gaussian curvature surfaces, a transverse curvature radius ($R_{T}$) of 800 mm is taken and the maximum compression ratios ($\lambda_{\max}$) are set as 5%, 6%, and 7%, respectively. In the case of negative Gaussian curvature surfaces, the maximum compression ratio is 5%, and the transverse curvature radii are 800 mm, 850 mm, and 900 mm, respectively. The simulated and corrected calculated profiles of the longitudinal centerline of the 3D surface are compared, as shown in Figure 17.

$Y_{C0}{}^{\prime }$ and $Y_{S0}{}^{\prime }$ are the calculated radii of the longitudinal centerline of the positive and negative Gaussian curvature surfaces after correction, while $Y_{C1}$ and $Y_{S1}$ are the corresponding simulated values. As can be seen in Figure 17, the corrected calculated and simulated profiles fit very well. The error of the longitudinal curvature radius is calculated to be no more than 8% for the positive Gaussian curvature surface and no more than 7% for the negative Gaussian curvature surface, indicating that the predicted formulas are also applicable to the 08 Al sheet, so it is considered that the formulas established in this paper can be applied to continuous roll forming with different materials.

## 5. Conclusions

Due to its ability to rapidly form double curvature surfaces with different shapes, continuous roll forming has wide application prospects. Ignoring the elastic deformation, the transverse curvature radius of the double curvature surface is the same as the radius of the centerline of the roll gap, and thus, the derivation of the longitudinal curvature radius becomes the key to shape prediction. In this paper, a new method for shape prediction of the sheet in CRF is proposed based on the analysis of exit velocity. The specific conclusions are as follows:

1. The critical aspect of CRF is that the rolls are curved and the roll gap is not constant in the transverse direction. The fundamental reason for the longitudinal bending of sheet metal is that the uneven roll gap causes the velocity difference of the exit cross-section at different positions in the transverse direction, which makes the exit cross-section rotate.
2. The calculation formulas for the longitudinal curvature radius are deduced, and it is found that the longitudinal radius of the positive Gaussian curvature surface is larger than the calculated value, and the longitudinal radius of the negative Gaussian curvature surface is smaller than the calculated value. It is analyzed that the error is caused by the longitudinal exit velocity not following the ideal linear distribution with respect to the Z-coordinate. The correction coefficients of the formulas based on Box–Behnken design are given as 1.138 and 0.905 for positive and negative Gaussian curvature surfaces, respectively.
3. The effects of maximum compression ratio, sheet width, sheet thickness, and transverse curvature radius on the longitudinal curvature radius are analyzed by adopting the response surface methodology. The results reveal that the sheet width and sheet thickness have positive effects, while the maximum compression ratio and transverse curvature radius have negative effects.
4. The validity of the corrected formulas is verified by numerical simulations and forming experiments. The numerical simulations show that the simulated profiles of the longitudinal centerlines fit well with the corrected calculated profiles. The experimental results indicate that the forming errors of the parts are very small. Compared with the predicted surfaces, the absolute values of the normal errors of the transverse profiles (x = 0 and x = ±70 mm) are within 3.5 mm for the positive Gaussian curvature surface and within 2 mm for the negative Gaussian curvature surface.
5. Numerical simulations are conducted to demonstrate that the predicted formulas for the longitudinal curvature radius are equally applicable to the 08 Al sheet, so it is considered that the formulas derived in this paper can be applied to the shape prediction of the sheet in continuous roll forming with different materials.

## Figures and Tables

**Table 1:** Chemical composition of Al 2024-O.

Chemical Element	Al	Cu	Mg	Mn	Cr	Fe	Si	Ti	Zn
**Content (%)**	90.7–94.7	3.8–4.9	1.2–1.8	0.3–0.9	0.1	0.5	0.5	0.15	0.25

**Table 2:** Levels and codes of factors in BBD.

Code	Level			
	X_1_	X_2_ (mm)	X_3_ (mm)	X_4_ (mm)
−1	0.04	210	1.5	800
0	0.06	240	1.75	1000
1	0.08	270	2	1200

**Table 3:** Test arrangement and results of BBD.

Run	X_1_	X_2_	X_3_	X_4_	$Y_{c0}\;(\mathrm{mm})$	$Y_{c1}\;(\mathrm{mm})$	$\Delta Y_{c}\;(\mathrm{mm})$	$Y_{s0}\;(\mathrm{mm})$	$Y_{s1}\;(\mathrm{mm})$	$\Delta Y_{s}\;(\mathrm{mm})$
1	0	0	−1	−1	159.90	169.55	9.65	150.85	114.85	36.00
2	0	−1	1	0	97.66	144.94	47.28	92.13	95.44	3.31
3	1	0	−1	0	97.55	101.67	4.12	90.33	65.32	25.01
4	0	−1	0	1	81.31	97.81	16.50	76.71	77.53	0.82
5	−1	1	0	0	238.01	250.80	12.79	228.86	208.33	20.53
6	0	0	−1	1	106.27	110.36	4.09	100.25	81.88	18.37
7	1	0	1	0	97.55	120.13	22.58	90.33	88.80	1.53
8	−1	−1	0	0	143.72	187.63	43.91	138.19	142.53	4.34
9	0	1	−1	0	161.73	164.11	2.38	152.57	114.51	38.06
10	−1	0	0	−1	235.33	256.88	21.55	226.28	206.11	20.17
11	−1	0	0	1	156.39	195.09	38.70	150.38	153.36	2.98
12	0	0	1	1	106.27	141.88	35.61	100.25	104.32	4.07
13	0	0	0	0	127.66	126.46	1.20	120.44	108.63	11.81
14	0	0	0	0	127.66	126.46	1.20	120.44	108.63	11.81
15	0	−1	−1	0	97.66	99.29	1.63	92.13	80.27	11.86
16	0	−1	0	−1	122.26	121.85	0.41	115.34	106.25	9.09
17	0	1	0	−1	202.69	225.29	22.60	191.21	152.87	38.34
18	0	1	0	1	134.58	164.39	29.81	126.97	107.11	19.86
19	0	0	0	0	127.66	126.46	1.20	120.44	108.63	11.81
20	1	1	0	0	123.58	139.56	15.98	114.43	83.12	31.31
21	−1	0	−1	0	187.88	225.79	37.91	180.65	161.37	19.28
22	−1	0	1	0	187.88	300.29	112.41	180.65	234.78	54.13
23	0	0	1	−1	159.90	195.65	35.75	150.85	149.58	1.27
24	0	1	1	0	161.73	172.24	10.51	152.57	134.33	18.24
25	1	−1	0	0	74.63	73.13	1.50	69.10	60.91	8.19
26	1	0	0	−1	122.19	136.85	14.66	113.14	86.04	27.10
27	1	0	0	1	81.20	80.35	0.85	75.19	64.12	11.07

**Table 4:** Arrangement of verification test.

Test Number	X_1_	X_2_ (mm)	X_3_ (mm)	X_4_ (mm)
1	0.05	225	1.8	950
2	0.055	231	1.9	1050
3	0.065	249	1.6	850

## Data Availability

The data that support the findings of this study are available from the corresponding author upon reasonable request.
